# Timing and intensity of changes in FDG uptake with symptomatic esophagitis during radiotherapy or chemo-radiotherapy

**DOI:** 10.1186/1748-717X-9-37

**Published:** 2014-01-27

**Authors:** Shuanghu Tiger Yuan, Richard KJ Brown, Lujun Zhao, Randall K Ten Haken, Milton Gross, Kemp B Cease, Matt Schipper, Paul Stanton, Jinming Yu, Feng-Ming Spring Kong

**Affiliations:** 1 Department of Radiation Oncology, University of Michigan, 1500 E. Medical Center Drive, Ann Arbor, MI 48109, USA; 2 Department of Radiation Oncology, Shandong Cancer Hospital and Institute, Jinan 250117 P.R. China; 3 Department of Radiology, Division of Nuclear Medicine, University of Michigan, Ann Arbor MI 48109, USA; 4 Department of Radiation Oncology, Tianjin Medical University Cancer Hospital, Tianjin 300060, China; 5 Department of Internal Medicine, University of Michigan, Ann Arbor, MI 48109, USA; 6 Department of Radiation Oncology, Veteran Administration Health Center, Ann Arbor, MI 48109, USA; 7 Department of Radiation Oncology, Lung Multidisciplinary Clinic, Lung and Esophageal Programs, GRU Cancer Center/Medical College of Georgia, Georgia Regents University, Augusta, GA 30912, USA

**Keywords:** Non-small cell lung cancer, FDG PET, Standard uptake value (SUV), Radiation esophagitis

## Abstract

**Purpose:**

To study whether esophageal FDG activity changes by time of mid-course of fractionated radiotherapy (RT), and whether these changes are associated with radiation esophagitis in patients with non-small cell lung cancer (NSCLC).

**Methods:**

Fifty patients with stage I-III NSCLC were enrolled prospectively and, all received ≥60 Gy RT. FDG-PET/CT scans were acquired prior to, and during-RT after delivery of 45 Gy. Normalized standardized uptake values (NSUV), defined by the esophageal maximum SUV relative to intravascular background level in the aortic arch, were sampled in the esophagus at the level of the primary tumor, sternal notch, aortic arch, carina, and gastro-esophageal junction. Symptomatic radiation esophagitis was defined as an event.

**Results:**

Compared to baseline, esophageal NSUV increased significantly during-RT at the level of the primary tumor (1.09 ± 0.05 vs.1.28 ± 0.06, p = 0.001), but did not change at other levels in the esophagus. 16 patients had radiation esophagitis events and these patients had significantly higher during-RT to baseline NSUV ratios than those without esophagitis (1.46 ± 0.12, 95% CI 1.20-1.71; vs. 1.11 ± 0.05, 95% CI 1.01-1.21, p = 0.002). Maximum esophageal dose (p = 0.029), concurrent chemotherapy (p = 0.022) and esophageal FDG PET NSUV ratio (during-RT to baseline, p = 0.007), were independent factors associated with esophagitis and area under curves (AUC) were 0.76, 0.70 and 0.78, respectively. Combining esophageal maximum dose and FDG PET NSUV Ratio at the tumor level increased AUC to 0.85 (p = 0.016).

**Conclusion:**

FDG uptake increased in esophagus during-RT and this increase may predict radiation esphagitis during later course of treatment.

## Introduction

Esophagitis is a common complication of patients who undergo thoracic radiation therapy (RT) for non–small cell lung cancer (NSCLC) and a source of considerable morbidity [1,2]. Patients often complain of dysphagia and/or odynophagia late during the course of fractioned radiotherapy. Severe esophagitis can necessitate hospitalization, initiation of percutaneous or parenteral feeding, and treatment interruption. These complications significantly affect quality of life and can negatively impact long-term survival [3].

Clinical and dosimetric studies predicting the risk of radiation esophagitis have been performed [4-6]. For example, concurrent chemotherapy and esophageal radiation dose were correlated with the clinical severity of esophagitis, in some reports [7,8]. ^18^Fluorodeoxyglucose-positron emission tomography (FDG-PET) can show the functional status of a tumor through the visualization of its metabolic activity gauged by FDG uptake, and it can also provide additional information to aid in the understanding of radiation induced-esophagitis. Some investigators have reported esophageal FDG uptake after radiation therapy [9-14]. However, the correlation of esophageal FDG uptake and clinical signs and symptoms of radiation induced-esophagitis were not well studied. The purpose of this study was to examine whether esophageal FDG activity changes by mid-course of fractionated RT, and whether these changes in esophageal FDG uptake are associated with radiation induced-esophagitis late during course of treatment in patients with NSCLC.

## Materials and methods

### Study population

The study population included consecutively enrolled patients in prospective studies of FDG PET to predict treatment outcomes, approved by the institutional review board of University of Michigan. Adult patients with histologically confirmed stage I to III NSCLC (AJCC 2003) undergoing definitive radiation with or without chemotherapy were enrolled in the study. Patients with small cell lung cancer or mixed small cell/non-small cell tumor histology, pericardial effusion, pregnant or lactating were excluded from the study. This study received ethical approval from the institution review boards of University of Michigan and Ann Arbor VA. Written informed consent was obtained from the patient for publication of this Case report and any accompanying images. A copy of the written consent is available for review by the Editor-in-Chief of this journal.

### FDG PET/CT imaging

The FDG-PET/CT scans were acquired 1–2 weeks before RT (pre-RT), and after the delivery of approximately 45 Gy (during-RT). The time of delivery of approximately 45 Gy of the total prescribed dose was chosen for the during-RT scan, with the intent that a dose to this threshold would provide control of microscopic disease and leave a reasonable amount of treatment remaining to alter the radiation therapy plan to include an additional RT boost. In cases of radiation administered in fractions other than 2 Gy, tumor dose was converted to a biological equivalent dose of approximately 45 Gy in 2-Gy fractions.

**Figure 1:**
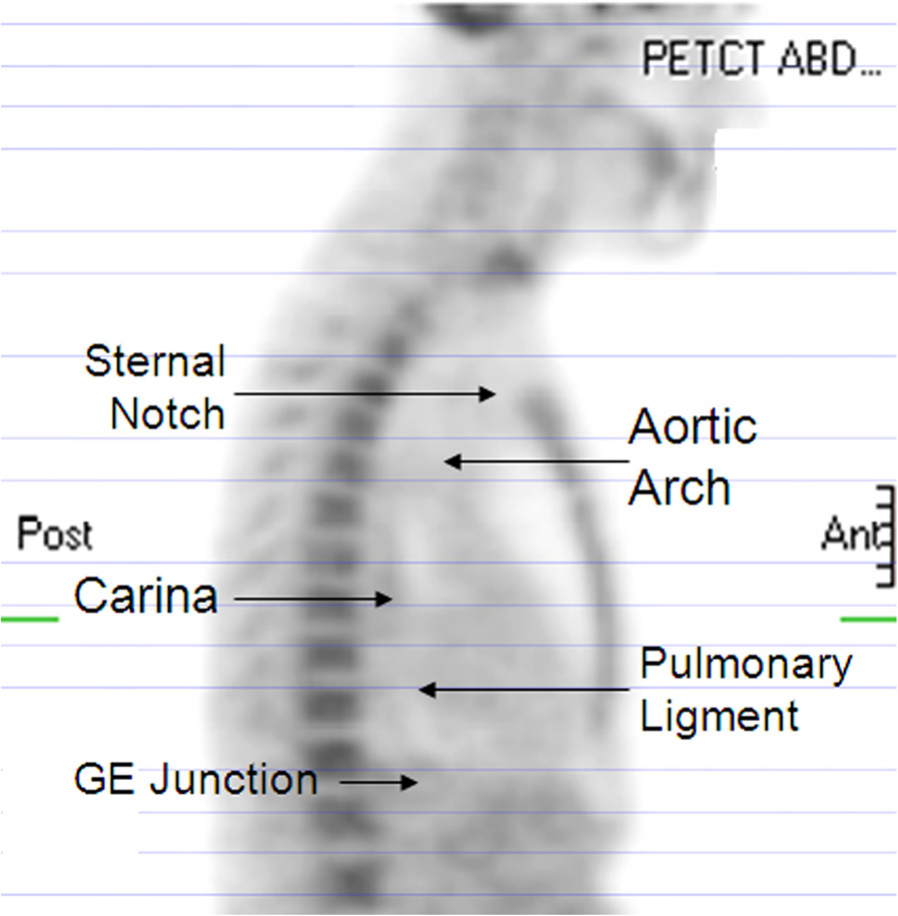
Esophageal uptakes of FDG were measured at tumor level and other levels marked.

The FDG-PET/CT scans were performed using a hybrid PET/CT scanner (Biograph Classic; Siemens Medical Solutions, Hoffman Estates, IL), with arms raised above the head in treatment position, as previously described [15]. Blood glucose levels were screened prior to PET and were less than 150 mg/ml. CT images (5-mm slices) for the PET/CT study were obtained during quiet ventilation. Emission PET images were obtained beginning 60 minutes after administration of 8 to 10 mCi of [^18^ F] FDG. PET scans were evaluated qualitatively by both a nuclear medicine physician (R.B.) and a radiation oncologist (S. Y.), and by objective quantification of FDG uptake in regions of interest (ROI). SUV estimation was performed using e.soft express (version 3.5; Siemens Medical Solutions). SUVmax activity, relative to intravascular background from the aortic arch, was measured in the esophagus at the level of the lung tumor level, the sternal notch, aortic arch, carina, gastric-esophageal junction and other normal tissues including axillary fat, liver, and erector muscle of spine (Figure 1). Given that SUV varies with many factors, such as the exact amount of radioactive tracer administrated, the interval between tracer injection and the scanning time, the blood glucose level, etc., normalized SUV was used (NSUV) to measure the FDG activity of each region of interest (ROI) to improve reproducibility. NSUV was calculated as: f: NSUV = SUV max of ROI/ mean SUV of the aortic arch [15]. SUVmax was chosen to measure the esophagus because it is the most reproducible measurement and a commonly used clinical parameter. Mean SUV is more representative of the activity of mediastinal background in the aortic arch where FDG uptake is more homogeneous and this is a standard area to determine background blood pool FDG activity.

### Esophagitis

Radiation-induced esophagitis, represented clinically by the presence of either dysphagia or odynophagia, was assessed weekly and graded prospectively according to the National Cancer Institution (NCI) Common Toxicity Criteria for Adverse Events (CTCAE), version 3.0. The maximum degree of esophagitis documented during the course of radiotherapy or follow-up was considered the study endpoint for each patient.

### Statistical consideration

The differences in FDG activity between time points were assessed by paired *t*-test and the difference between patients with and without symptomatic radiation esophagitis was assessed by independent *t*-test. A binary Logistic regression analysis model was used to test the correlations between symptomatic radiation-induced esophagitis, FDG activity ratio change and clinical findings. The performance of predictive models for esophagitis was analyzed by area under receiver operating characteristic curve (AUC).

## Results

### Patient characteristics and their relationship with radiation esophagitis

A total of 50 patients with stage I-III NSCLC requiring radiation therapy were enrolled from 2003 to 2009. Minimum follow-up was 24 months for surviving patients. All patients had FDG PET/CT scans within 1–2 weeks prior to the initiation of radiation therapy and after delivery of approximately 45 Gy (during-RT). Patient demographics are shown in Table 1. Twenty-three (46%) patients had medically inoperable stage I–II disease and the remaining 27 (54%) patients had locally advanced stage IIIA–B disease. Thirty-three patients (66%) received concurrent chemo-radiotherapy, while the remaining patients received definitive radiation alone. The average mean radiation dose to tumor was 68.0 (95% CI 65.0-71.3) Gy while the average mean and maximum radiation dose to the esophagus was 22.1 (95% CI 17.9-26.3) and 55.1 (95% CI 48.5-61.6) Gy, respectively.

**Table 1:** Patient characteristics and symptomatic esophagitis

	**Total patients**	**Patients without esophagitis**	**Patients with esophagitis**	***P***	
				**UV**	**MV**
Gender				0.699	
Male	41	27	14		
Female	9	7	2		
Median Age	68	71	65	0.044	>0.05
(95% CI)	(61–75)	(68–73)	(60–71)		
Clinical stage				0.032	>0.05
I	14	12	2		
II	9	8	1		
III	27	14	13		
Histology				0.163	
Adenocarcinoma	6	3	3		
Squamous carcinoma	11	10	1		
NSCLC, unspecified	33	21	12		
Concurrent chemotherapy				0.004	0.022
With	33	18	15		
Without	17	16	1		
Mean tumor dose	68.0	66.2	66.2	0.380	
(95% CI, Gy)	(65.0–71.3)	(63.6–68.9)	(65.3–70.9)		
Maximal esophageal dose	55.1	47.9	69.0	0.001	0.029
(95% CI, Gy)	(48.5–61.6)	(39.0–56.8)	(65.5–72.4)		
Esophagus FDG uptake change at tumour level (During-RT/pre-RT)	1.22	1.11	1.46	0.002	0.007
	(1.11–1.33)	(1.01–1.21)	(1.20–1.71)		

*Abbreviations*: *CI* confidence interval, *UV* univariate analysis, *MV* multivariate analysis.

Sixteen of 50 (32%) patients had clinically apparent radiation-induced esophagitis. These patients were younger than patients without symptomatic esophagitis (Median age 65 vs. 71, p = 0.044). Patients with stage III NSCLC had a higher rate of radiation-induced esophagitis than those with stage I-II (48% vs. 13%, p = 0.032). Patients treated with concurrent chemotherapy had a higher rate of esophagitis than those not treated with concurrent chemotherapy (45% vs. 6%, p = 0.004). Patients with esophagitis also had higher maximum esophageal radiation dose (69.0 Gy vs. 47.9 Gy, p = 0.001) than patients without esophagitis (Table 1). There were no differences in gender, KPS, histology type and prescribed PTV dose between patients with or without esophagitis.

### FDG-uptake and esophagitis

**Figure 2:**
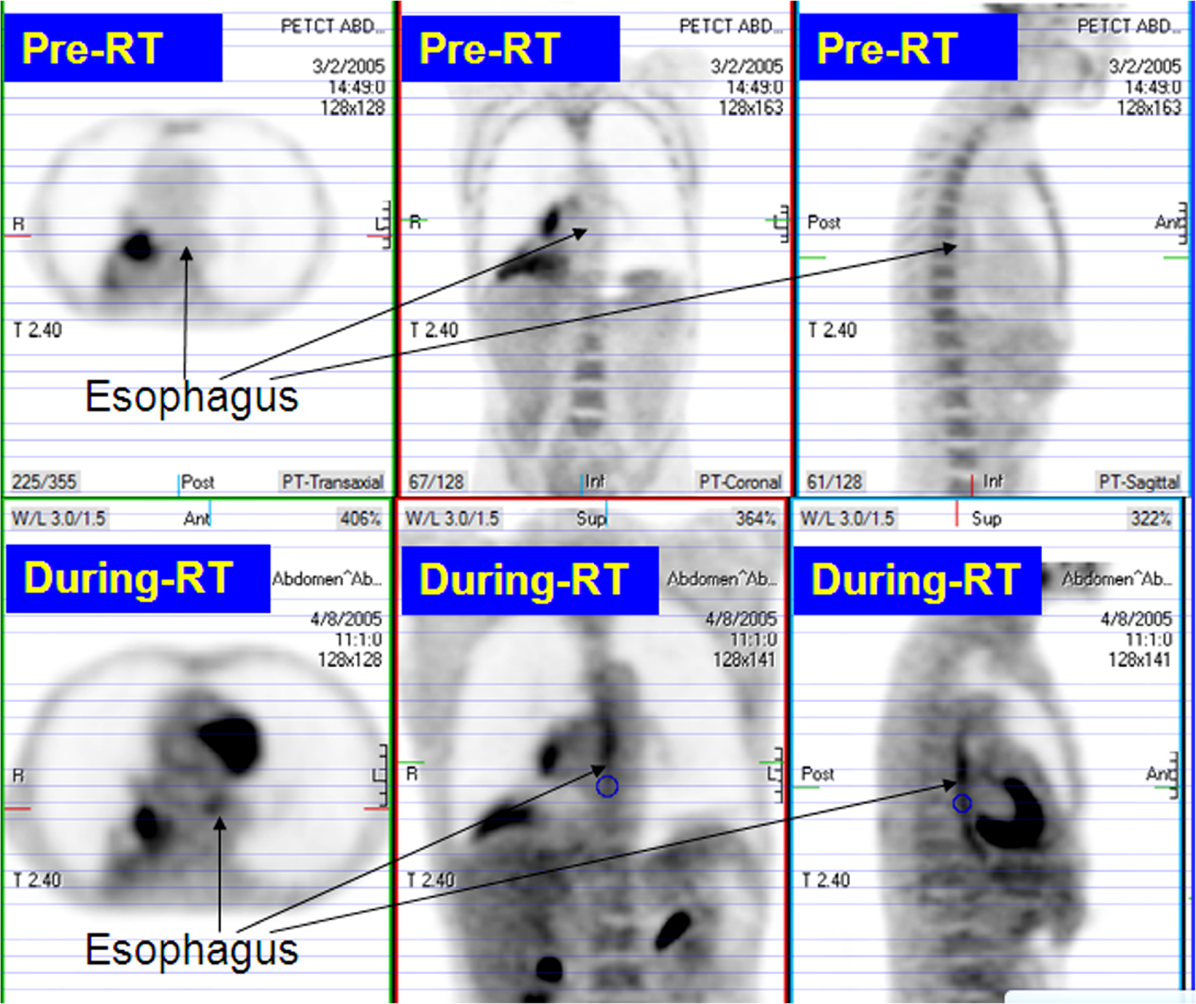
**FDG-PET scans in a patient with symptomatic esophagitis.** This patient received 66 Gy and required G-tube in 1 month after completion of radiotheapy.

**Figure 3:**
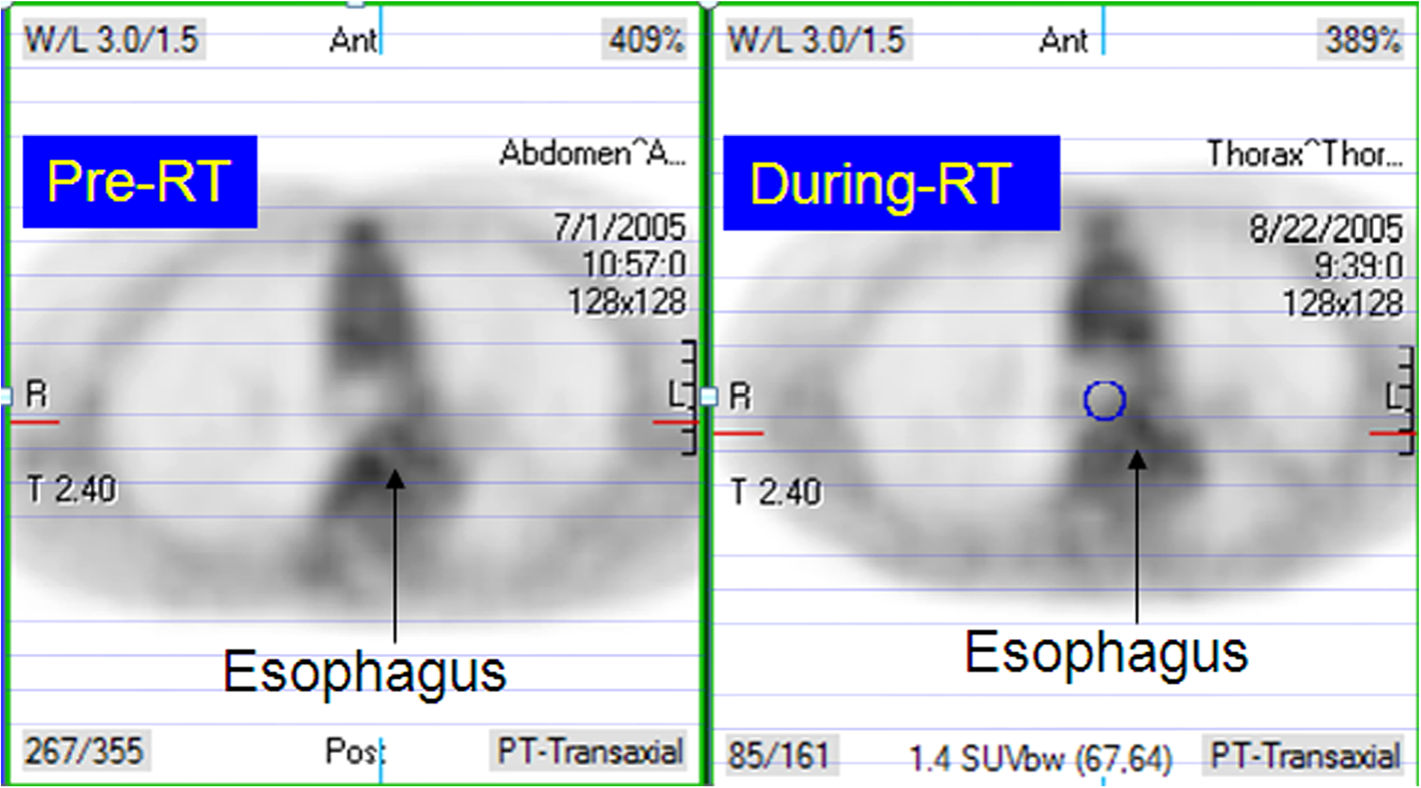
FDG-PET scans in patient without symptomatic esophagitis.

NSUV of esophagus pre-RT and during-RT FDG PET are summarized in Table 2. No significant NSUV change between pre-RT scan and during-RT scan was observed in auxiliary fat (0.24 vs. 0.25, p = 0.429), liver (1.43 vs. 1.33, p = 0.157) and prespinal muscle (0.51 vs. 0.43, p = 0.217). Compared to baseline, esophageal NSUV increased significantly during-RT at the level of the tumor (1.09 ± 0.05 vs. 1.28 ± 0.06, p = 0.001), but did not change significantly elsewhere (Figures 2 and 3).

**Table 2:** Changes of relative FDG uptake during the course of radiotherapy

**FDG uptake (NSUV)**	**Pre-RT**		**During-RT**		***P***
	**Mean**	**95% CI**	**Mean**	**95% CI**	
Esophagus					
Sternal notch level	1.03	0.94–1.17	1.07	0.96–1.18	0.572
Aortic arch level	1.03	0.96–1.13	1.11	1.01–1.22	0.139
Carina level	1.17	1.05–1.28	1.25	1.12–1.37	0.244
Pulmonary ligament level	1.16	1.04–1.27	1.28	1.15–1.41	0.168
GE junction	1.27	1.12–1.39	1.39	1.24–1.56	0.189
Tumour level	1.09	1.00–1.18	1.28	1.17–1.39	0.001
Axillary fat	0.24	0.21–0.26	0.25	0.21–0.28	0.429
Liver	1.43	1.34–1.49	1.33	1.20–1.46	0.157
Erector muscle of spine	0.51	0.37–0.60	0.43	0.36–0.50	0.217

*Abbreviation*: *NSUV* normalized standard uptake value. All the FDG uptake was normalized to that of aortic arch.

Patients with esophagitis had a significantly higher during-RT to baseline NSUV ratio at the tumor level than those without (1.46 [95% CI 1.20-1.71] vs. 1.11 [95% CI 1.01-1.21], p = 0.002).

### Multivariate analysis for esophagitis

**Figure 4:**
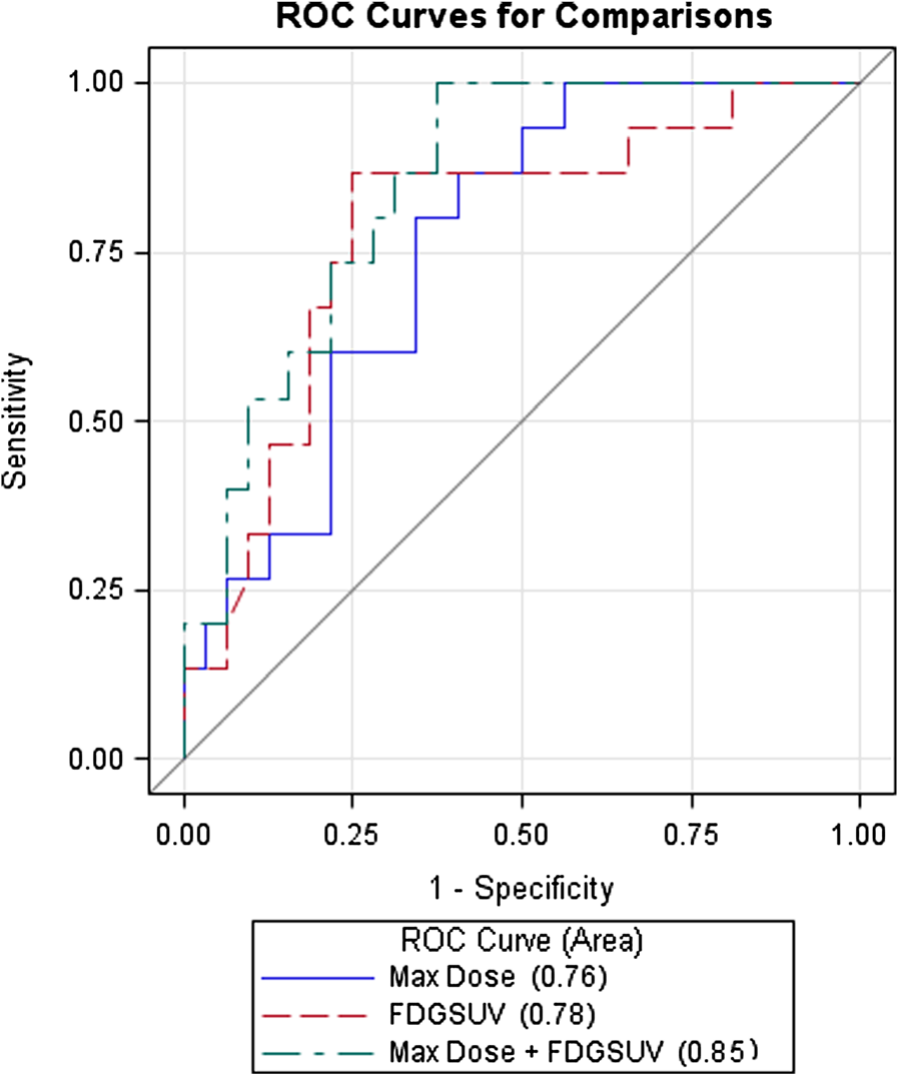
Receiver operating characteristic (ROC) curve comparision.

All significant univariate analysis factors were entered into multivariate logistic regression analysis. Maximum esophageal dose (p = 0.029), concurrent chemotherapy (p = 0.022) and esophageal FDG PET NSUV (during-RT to baseline, p = 0.007), were independent factors associated with esophagitis (Table 1) with AUCs of 0.76, 0.70 and 0.78, respectively, under receiver operating characteristic (ROC) curve analysis. Combining esophageal maximum dose and FDG PET NSUV ratio at the tumor level increased AUC to 0.85 (p = 0.016, Figure 4).

## Discussion

We analyzed the FDG PET scans of 50 patients with NSCLC and found that esophageal NSUV at the tumor level increased during-RT. NSUV increase from pre-RT is an independent measurement related to radiation-induced esophagitis, reflecting the accumulation of FDG in affected esophageal mucosa. Thus, FDG PET SUV may be an important supplement to traditional dose volume parameters associated with radiation-induced esophagitis.

Although there have been reports on the relationship of radiation esophagitis and FDG-PET, to our knowledge, this is the first study documenting the correlation between during-RT esophageal FDG-uptake and the presence of clinically documented radiation-induced esophagitis [9-14]. This is in addition to reports of patients with FDG uptake from bacterial esophagitis, Barrett’s esophagus or gastroesophageal reflux [16]. These conditions are characterized by linear intense FDG uptake throughout the esophagus not related to concurrent radiotherapy. The majority of radiation esophagitis reports using FDG PET are from esophageal cancer treatment [17,18]. Bhargava et al. demonstrated increased esophageal FDG activity that can be attributed to radiation therapy as radiation esophagitis in a patient with NSCLC. This diagnosis was based on post-RT FDG PET imaging and demonstrated by endoscopic esophageal biopsy [17]. Nijkamp et al. demonstrated that esophageal FDG uptake around 3 months after concurrent chemo-radiotherapy was associated with grade of esophagitis, and that there was a local dose–effect relationship with post-treatment FDG uptake, in which dose levels >55 Gy were indicative of increased FDG uptake [19]. However, a common factor of all of these prior studies is their usage of post-RT FDG PET. Our study of FDG-uptake during-RT provides the opportunity to detect the radiation esophagitis objectively on PET scan during-RT, which may predict radiation esophagitis in the clinic. Furthermore, during-RT assessments may guide radiation planning in each individual patient to esophagus sparing adaptive radiation therapy late during course. Patients with intense FDG-uptake in esophagus during-RT are at high risk for radiation esophagitis and further radiation to esophagus should be minimized in late course radiotherapy.

FDG activity may be used to supplement traditional dose volume parameters associated with radiation-induced esophagitis. In this study, we found that esophageal FDG SUV increase, concurrent chemotherapy and maximal radiation dose to the esophagus were independent factors associated with the development of radiation esophagitis.

## Competing interests

The authors declare that they have no competing interests.

## Authors’ contributions

SY and RB evaluated the PET scans and SY carried out the data collection, analysis and writing. LZ and MG participated in the methodology development of PET scans evaluation. RTH participated in study design, data analysis and writing. KC graded the esophagitis. MS carried out the statistics. PS participated in the data collection and writing. JY participated in study design and writing. FK designed, supervised and supported this study. All authors read and approved the final manuscript.
